# Expression Pattern of Transcription Factors and Intracellular Cytokines Reveals That Clinically Cured Tuberculosis Is Accompanied by an Increase in *Mycobacterium*-Specific Th1, Th2, and Th17 Cells

**DOI:** 10.1155/2015/591237

**Published:** 2015-04-27

**Authors:** Marcos V. da Silva, Vladimir J. Massaro Junior, Juliana R. Machado, Djalma A. A. Silva, Lúcio R. Castellano, Patricia B. D. Alexandre, Denise B. R. Rodrigues, Virmondes Rodrigues

**Affiliations:** ^1^Laboratory of Immunology, Institute of Biological Sciences, Triângulo Mineiro Federal University, 38025-180 Uberaba, MG, Brazil; ^2^Department of Pathology, Institute of Tropical Pathology and Public Health, Federal University of Goiás, 74605-050 Goiania, GO, Brazil; ^3^Human Immunology Research and Education Group (GEPIH), Technical School of Health, Federal University of Paraíba, 58051-900 João Pessoa, PB, Brazil; ^4^Municipal Secretary Office of Health, 38065-160 Uberaba, MG, Brazil; ^5^Laboratory of Biopathology and Molecular Biology, University of Uberaba, 38055-500 Uberaba, MG, Brazil

## Abstract

Tuberculosis (TB) remains a major global health problem and is the second biggest cause of death by infectious disease worldwide. Here, we investigate in vitro the Th1, Th2, Th17, and Treg cytokines and transcriptional factors produced after* Mycobacterium*-specific antigen stimulation in patients with active pulmonary tuberculosis, clinically cured pulmonary tuberculosis, and healthy donors with a positive tuberculin skin test (TST+). Together, our data indicate that clinical cure after treatment increases the percentages of* Mycobacterium*-specific Th1, Th2, and Th17 cells compared with those found in active-TB and TST+ healthy donors. These results show that the host-parasite equilibrium in latent TB breaks in favor of the microorganism and that the subsequent clinical recovery posttreatment does not return the percentage levels of such cells to those observed in latent tuberculosis. Additionally, our results indicate that rather than showing an increase in the percentage of* Mycobacterium*-specific Tregs, active-TB patients display lower Th1 : Treg and Th17 : Treg ratios. These data, together with lower Th1 : Th2 and Th17 : Th2 ratios, may indicate a mechanism by which the breakdown of the host-parasite equilibrium leads to active-TB and changes in the repertoire of* Mycobacterium*-specific Th cells that are associated with clinical cure after treatment of pulmonary tuberculosis.

## 1. Introduction

Tuberculosis (TB) remains a major global health problem and is the second biggest cause of death by infectious disease worldwide. According to the World Health Organization (WHO), approximately one-third of the world's population is infected by* Mycobacterium tuberculosis* (Mtb) and approximately 8.6 million new TB cases and 1.6 million deaths occur per year [1, 2]. It is estimated that one-third of the world's population is infected with Mtb and 90–95% of those infected are asymptomatic; this status is called latent TB [1]. Of those infected, 5–10% progress to active disease [3]. In the case of chronic infection, most infected individuals maintain a lifelong state of latency. Several immune mechanisms have been proposed regarding the maintenance of this latency, especially those related to cellular immune response development and T helper-derived cytokine profile, including macrophage activation and granuloma maintenance [4].

CD4+Th1 cells are interferon-gamma (IFN-*γ*) producers and their signature transcription factor is T-box expressed in T cells (T-bet). Previous results from our group point to an increase in IFN-*γ* production that is associated with clinically cured TB, although the IFN-*γ* source still needs to be determined [5]. Additionally, mice are incapable of controlling a low-dose Mtb infection in the absence of IFN-*γ* [6]. On the other hand, the Th2 cell profile is characterized by IL-4 and IL-13 production and expression of GATA-binding protein-3 (GATA-3), a transcription factor that acts in a regulatory feedback loop to further increase IL-4 and IL-13 production [7, 8]. A Th2 profile-predominant response has been associated with susceptibility to TB, due to IL-12R and STAT4 inhibition by GATA-3, weakened Th1-mediated immunity, and IL-4- and IL-13-induced alternative macrophage activation and inhibition of autophagic control of Mtb [8, 9]. This association persists, even though several studies, especially in humans, have failed to clearly show this relationship at the single-cell level, due to solely evaluating cytokine production or plasma levels [10–13], thus making it difficult to determine whether they are the cause or the consequence of infection reactivation [14]. Th17 cells, which produce IL-17 and IL-22, contribute to immune defense against Mtb by cytokine and chemokine induction of initial neutrophil recruitment and granuloma formation [15]. Naïve T cell activation, in the presence of transforming growth factor-*β* (TGF-*β*) and IL-6, leads to initial Th17 cell differentiation, and STAT3 activation by these cytokines upregulates the RAR-related orphan receptor *γ*-t (ROR*γ*t) transcriptional factor, while both factors increase proinflammatory IL-17 cytokine production [16]. However, studies show that IL-17 overproduction may induce pathological effects during Mtb-induced inflammation [17], although its role in establishing clinically cured TB in humans still needs to be determined. Treg cells are essential for immune tolerance and can suppress the effector activity of several other Th subsets [18]. These cells are characterized by expression of the transcriptional factor forkhead box protein 3 (Foxp3) and may express high levels, low levels, or no IL-2 receptor *α*-chain (CD25); yet they still reveal the same transcriptional signature and potent suppressor function [19]. In human tuberculosis, the role of T regulatory cells remains controversial, especially the relationship between protective and pathological responses [20–22].

In our study, we used a single-cell analysis approach to investigate the* in vitro* percentage levels of Th1, Th2, Th17, and Treg cells after* in vitro Mycobacterium-*specific antigen stimulation in active pulmonary tuberculosis, clinically cured pulmonary tuberculosis, and TST+ healthy donors to determine the extent to which active disease development, therapeutic intervention, and clinical recovery impact the percentage levels of these cells. Our results suggest that the development of active pulmonary tuberculosis reduces the percentage of* Mycobacterium*-specific Th1 cells but not Th2 cells, which become predominant, compared with Th1 and Th17 cells. Additionally, active-TB patients have a predominance of various subpopulations of potential regulatory cells (FoxP3+) compared with Th1 and Th17 cells. Moreover, the establishment of clinically cured TB leads to an overall increase in subpopulations of CD4+ T cells (Th1, Th2, Th17, and Treg), despite Th1 and Th17 cells predominate.

## 2. Patients and Methods

Peripheral blood samples were collected from 10 patients with active pulmonary tuberculosis (TB-active group), 10 patients with clinically cured pulmonary tuberculosis (TB-treated group), and a control group of 10 healthy individuals with no history of tuberculosis (pulmonary or extrapulmonary) but with a positive tuberculin skin test (healthy control), located in the same microregion and in compliance with the exclusion criteria. None of the TB-treated patients had any symptoms indicating that active disease developed after the end of treatment. All of the participants in this study were recruited from the city of Uberaba, in the state of Minas Gerais, in the southeast region of Brazil. Brazil is one of the 22 high-burden countries that collectively account for approximately 80% of the TB cases in the world. In the state of Minas Gerais, an average of 6,085 cases/year occurred in the last six years, with an incidence coefficient of 23 cases/100,000 inhabitants, the 4th highest TB burden in the country. The city of Uberaba had 73 confirmed cases in 2012 [23, 24].

The diagnosis of tuberculosis was based on clinical, radiographic, and laboratory findings, and it was performed by the Municipal Department of Health team in Uberaba, Minas Gerais state, Brazil. Patients with a diagnosis of pulmonary tuberculosis were immediately referred for specific chemotherapy according to the regimen recommended by the Brazilian Ministry of Health. Blood was collected from patients with active disease for a maximum period of 21 days after the beginning of treatment in order to reduce the impact of therapy on the parameters studied. HIV-infected patients (regardless of clinical disease status), transplant patients, patients using immunosuppressive drugs, patients with chronic alcoholism, malnourished patients, and patients with any known cause of immunosuppression were excluded from the study. All of the participants recruited were previously vaccinated with BCG.

### 2.1. Ethics Statement

This study was approved by the Ethics Committee of Federal University of Triângulo Mineiro, Uberaba, Minas Gerais state, Brazil, and all participants signed the Free and Informed Consent Form.

### 2.2. Isolation and Culture of Peripheral Blood Mononuclear Cells

Peripheral blood mononuclear cells (PBMCs) were isolated by Ficoll-Hypaque density gradient centrifugation (GE Healthcare, Uppsala, Sweden) at 400 ×g at 21°C for 30 min. The cells were then resuspended in RPMI 1640 medium (GE Healthcare) containing 50 mM HEPES buffer (Gibco, Grand Island, NY, USA), 10% inactivated fetal bovine serum (Gibco), 2 mM L-glutamine (Gibco), 50 mM *β*-mercaptoethanol (Gibco), and 40 *μ*g/mL gentamicin (Neoquímica, Anápolis, GO, Brazil) to a final concentration of 2 × 10^6^ cells/mL. PBMCs were cultured in 24-well microplates (Falcon, San Jose, CA, USA) in the presence of 4 *μ*g/mL* M. bovis* antigen or maintained in culture medium at 37°C in a 5% CO_2_ atmosphere. The cells were collected after 48 h for immunophenotyping.

### 2.3. Preparation of* Mycobacterium bovis* Soluble Antigens

*Mycobacterium* antigens were extracted from* Mycobacterium bovis *(bacillus Calmette-Guérin (BCG)), strain Moreau (Instituto Butantan, São Paulo, Brazil). The mycobacteria were first resuspended in 0.85 g NaCl at a concentration of 2 × 10^6^ bacteria/mL, in accordance with the recommendations of Instituto Butantan. Next, the bacteria were incubated in a water bath at 90°C for 30 min and then autoclaved for 30 min. The cultures were centrifuged at 10,000 ×g at 4°C for 30 min and the supernatant (protein fraction) was collected and filtered through a 0.22 *μ*m filter (Millipore, Molsheim, France). Protein concentration was quantified by the Bradford method (Pierce, Rockford, IL, USA) and the protein fraction was divided into aliquots and stored at −20°C.

### 2.4. Analysis of Transcription Factors and Intracellular Cytokines Associated with T Helper Subsets

For analysis of T cell profiles, PBMCs cultured for 48 h were resuspended (5 × 10^5^ cells/mL) in Hank's medium (Sigma, St. Louis, MO, USA), washed three times (400 ×g, 4°C, 10 min), and incubated in Hank's medium supplemented with 10% inactivated human AB+ serum. Subsequently, the samples were labeled with corresponding surface antibodies for each T cell profile. The cells were washed to remove excess antibody and then permeabilized and fixed with the addition of 500 *μ*L Cytoperm/Cytofix (BD Biosciences, San Jose, CA, USA). Next, the samples were labeled with corresponding intracellular antibodies for each T cell profile and washed again to remove excess antibodies. Finally, the cells were resuspended in 500 *μ*L PBS containing 0.5% paraformaldehyde and stored at 4°C in the dark until flow cytometry analysis. The samples were labeled with antibodies (BD Biosciences, San Jose, CA, USA) for T cell profiles, including Th1 (IFN-*γ*-FITC, T-bet-PE, and CD4-PerCP), Th2 (IL-4-FITC, GATA-3-PE, and CD4-PerCP), Th17 (IL-17-FITC, ROR*γ*T-PE, and CD4-PerCP), and Treg (CD25-FITC, FoxP3-PE, and CD4-PerCP). A FACSCalibur cytometer (Becton-Dickinson, Mountain View, CA, USA) was used for the acquisition of events (100,000 events/tube) and the data were analyzed using the CellQuest program (Becton-Dickinson).

### 2.5. Statistical Analysis

Statistical analysis was performed using StatView software (version 4.57, Abacus Concept, Berkeley, CA, USA) and GraphPad Prism software (version 6.00, GraphPad Software, La Jolla, California, USA). Variables without normal distribution, which were expressed as the median, with range and percentiles, were analyzed using the Kruskal-Wallis test followed by Dunn's post hoc test to compare the three groups. Correlation analyses were performed using the Spearman test. Differences were considered statistically significant if *P* < 0.05.

## 3. Results

### 3.1. Percentage of* In Vitro* Activated CD4+ T Cells after Mycobacterial Antigen Stimulation Is Increased in TB-Treated Patients

**Figure 1:**
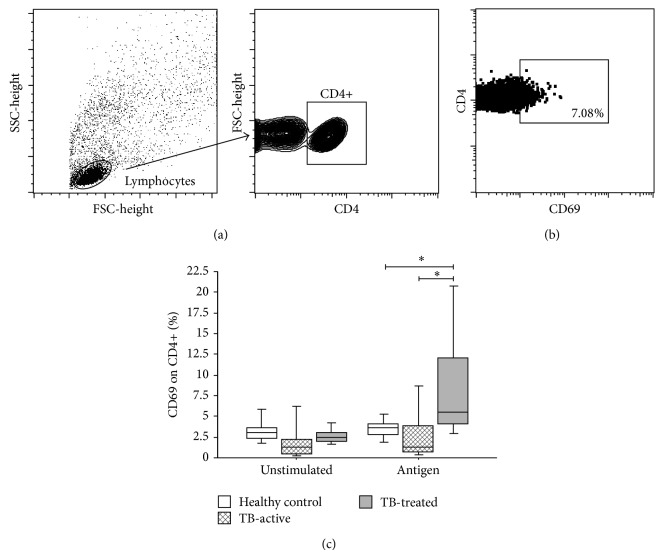
Activation of T helper cells in patients with active tuberculosis, clinically cured tuberculosis, and TST+ healthy donors. (a) Schematic representation of the gating strategy and determination of percentage of CD69+ cells among CD4+ cells in stimulated (4 *μ*g/mL* M. bovis* antigen) and unstimulated (medium only) cultures. First, T cells were separated based on FSC and SSC patterns. Then, these cells were isolated according to CD4 expression. (b) Activation was evaluated based on CD69 expression. Positive cells were defined using an appropriate isotype control. (c) Comparison among healthy donors TST+ (white box-plots), active-TB patients (dashed box-plots), and TB-treated patients (gray box-plots) in stimulated (4 *μ*g/mL* M. bovis* antigen) and unstimulated (medium only) cultures. ^*^ *P* < 0.05: Kruskal-Wallis test followed by Dunn's post hoc test. Horizontal lines represent the median, bars represent 25th–75th percentiles, and vertical lines represent 10th–90th percentiles.

The percentage of activated CD4+ T cells after specific stimulation was measured by the expression of CD69. CD69 is a molecule expressed in T cells after stimulation via the TCR [25, 26], and although it is an early activation marker that is expressed within minutes after cell activation, it is relatively stable, especially* in vitro* [27, 28]. Activated T cell phenotype was examined by the coexpression of CD4 and CD69 (activated T helper cells), as illustrated in Figures 1(a) and 1(b). In our study, we observed an increase in CD4+ T cell activation in the Pulmonary-TB-treated group in comparison with the active-pulmonary-TB group and TST+ healthy donors after antigen stimulation (*P* = 0.016 and *P* = 0.029, resp., Kruskal-Wallis test followed by Dunn's post hoc test) (Figure 1(c)).

### 3.2. Clinically Cured Tuberculosis Increases* Mycobacterium-Specific* T Helper Cells

**Figure 2:**
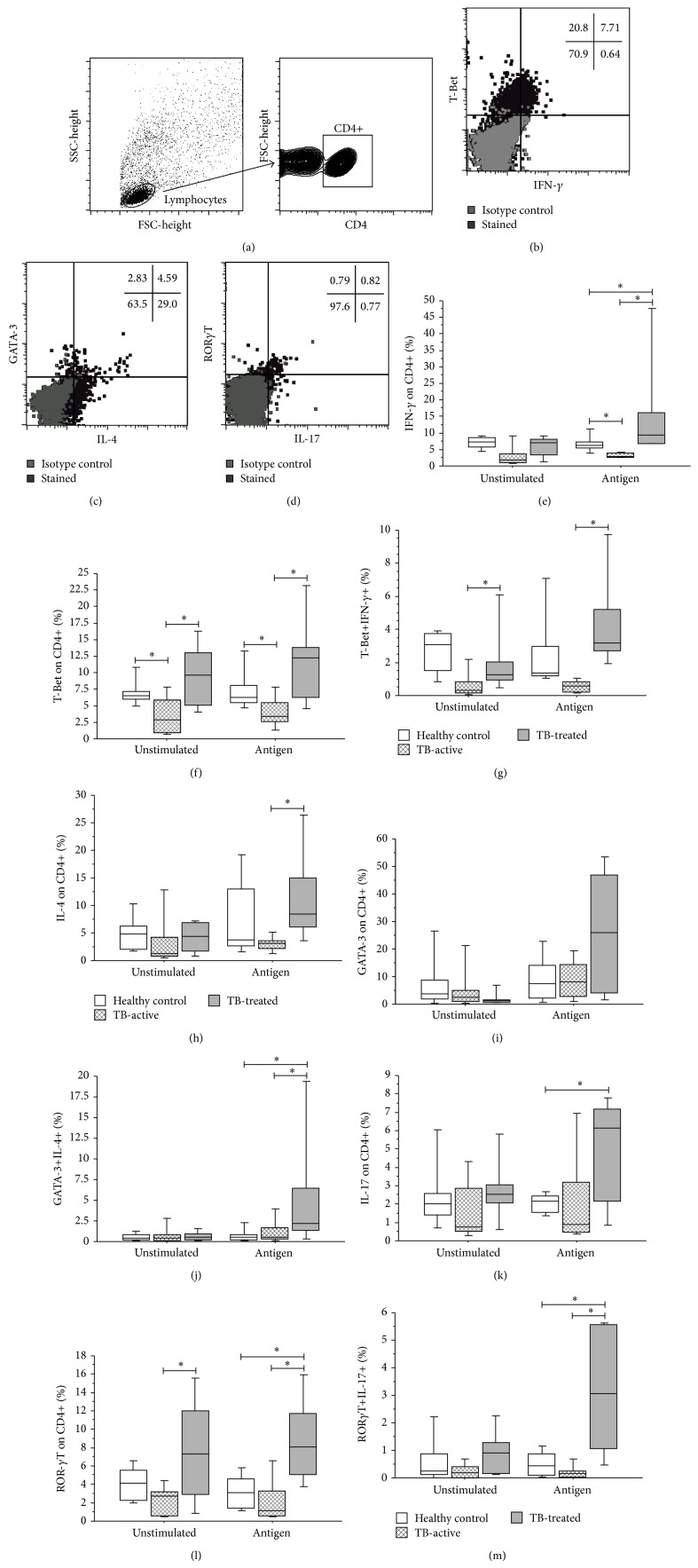
Transcription factors and cytokines in T helper cells from active tuberculosis patients, clinically cured tuberculosis patients, and TST+ healthy donors. (a) Schematic representation of the gating strategy of CD4+ T cells and determination of percentage of transcription factors and cytokines ((b) T-Bet and IFN-*γ*; (c) GATA-3 and IL-4; (d) ROR*γ*T and IL-17) in CD4+ cells. From left to right, T cells were separated based on FSC and SSC patterns. These cells were isolated according to CD4 expression and the expression of transcription factors and cytokines was evaluated. Dot plots representative of a single participant are shown. (e)–(m) Comparison of cytokines and transcription factors among TST+ healthy donors (white box-plots), active-TB patients (dashed box-plots), and TB-treated patients (gray box-plots) in stimulated (4 *μ*g/mL* M. bovis* antigen) and unstimulated (medium only) cultures. (e) %IFN-*γ*+; (f) %T-Bet+; (g) T-Bet+IFN-*γ*+; (h) %IL-4+; (i) %GATA-3+; (j) GATA-3+IL-4+; (k) %IL-17+; (l) %ROR*γ*T+; (m) ROR*γ*T+IL-17+. ^*^ *P* < 0.05: Kruskal-Wallis test followed by Dunn's post hoc test. Horizontal lines represent the median, bars represent 25th–75th percentiles, and vertical lines represent 10th–90th percentiles.

We evaluated CD4+ T cells expressing transcription factors and cytokines related to Th1 (T-Bet and IFN-*γ*), Th2 (GATA-3 and IL-4), and Th17 (ROR*γ*T and IL-17) after* in vitro *culture in the absence or presence of antigen stimulation. The results were expressed as the percentage of positive cells compared with CD4+ T cells, as illustrated in Figures 2(a)–2(d). We have observed, in general, that the clinical cure of pulmonary tuberculosis leads to an increase in Th1, Th2, and Th17* Mycobacterium*-specific cells compared with patients with active pulmonary tuberculosis and TST+ controls.

The pulmonary-TB-treated group showed a higher percentage of CD4+IFN-*γ*+ cells in antigen-stimulated cultures than did the active-pulmonary-TB group and healthy donors (*P* = 0.006 and *P* = 0.049, resp., Kruskal-Wallis test followed by Dunn's post hoc test). Furthermore, although TST+ healthy controls showed a lower percentage of antigen-specific CD4+IFN-*γ*+ cells than did clinically cured patients, they showed a higher percentage of these cells than did the active-pulmonary-TB group, but not statistically significantly (*P* = 0.04, Kruskal-Wallis test followed by Dunn's post hoc test), Figure 2(e).

Similarly, the percentage of CD4+T-bet+ cells was higher in clinically cured individuals with pulmonary tuberculosis than in those with active disease, both in cultures without stimulation (*P* = 0.001, Kruskal-Wallis test followed by Dunn's post hoc test) and with stimulation (*P* = 0.001, Kruskal-Wallis test followed by Dunn's post hoc test). Analogous to the CD4+IFN-*γ*+ cells, the healthy donors had a significantly higher percentage of antigen-specific CD4+T-bet+ cells than did those with active-pulmonary TB (unstimulated, *P* = 0.02; antigen, 0.021, Kruskal-Wallis test followed by Dunn's post hoc test), Figure 2(f). In evaluating the cells coexpressing T-bet and IFN-*γ* (classical Th1 cells), we observed an increase in the percentage of double-positive cells in clinically cured individuals when compared with unstimulated and stimulated cultures from the active disease group (*P* = 0.004 and *P* = 0.0006, resp., Kruskal-Wallis test followed by Dunn's post hoc test), Figure 2(g).

Regarding Th2 responses, we observed that the TB-treated patients had a significantly higher percentage of CD4+IL-4+ cells than did those presenting active disease (*P* = 0.005, Kruskal-Wallis test followed by Dunn's post hoc test), although this difference was not statistically significant when analyzing the isolated expression of GATA-3 (Figures 2(h) and 2(i)). However, we demonstrated a significantly higher percentage of CD4+GATA-3+IL-4+ cells in antigen-stimulated cultures from TB-treated patients, compared with those from TST+ controls and active-TB patients (*P* = 0.021 and *P* = 0.034, resp., Kruskal-Wallis test followed by Dunn's post hoc test), Figure 2(j).

Additionally, when analyzing the Th17-axis, we observed a significant increase in CD4+IL-17+ and CD4+ROR*γ*T+ cells in stimulated cultures from TB-treated patients compared with TST+ controls (*P* = 0.02 and *P* = 0.002, resp., Kruskal-Wallis test followed by Dunn's post hoc test), Figures 2(k) and 2(l). However, when TB-treated patients were compared to TB-Active patients, this difference was only significant for CD4+ROR*γ*T+, although under both stimulated and unstimulated culture conditions (*P* = 0.002 and *P* = 0.0003, resp., Kruskal-Wallis test followed by Dunn's post hoc test), Figure 2(l). In evaluating CD4+ROR*γ*T+IL-17+, we observed an increase in the percentage of antigen-specific cells in TB-treated patients compared with the active-TB patients and TST+ controls (*P* < 0.0001 and *P* < 0.0001, resp., Kruskal-Wallis test followed by Dunn's post hoc test).

Together, our data indicate that the processes of development of active pulmonary tuberculosis and subsequent clinical cure after treatment have a direct impact on the repertoire of T helper cells, and the process of clinical cure has an impact of increasing the percentage of* Mycobacterium*-specific Th1, Th2, and Th17 cells. Furthermore, despite the fact that all patients had been vaccinated during infancy with BCG and were TST+, the repertoire against soluble crude antigens of* Mycobacterium bovis* varies considerably, showing that the host-parasite equilibrium in latent TB breaks in favor of the microorganism and that subsequent clinical recovery posttreatment does not return cell percentage levels to those observed in latent tuberculosis.

### 3.3. Different T Regulatory Subsets Are Increased between Active-TB and Clinically Cured Tuberculosis Patients

Analogous to our observation of significant differences in the percentage of Th1, Th2, and Th17 cells among active-TB, TB-treated, and TST+ healthy donors, it is possible that these different clinical conditions affect the repertoire of T regulatory cells. Due to the wide phenotypic range described for these cells, we chose to evaluate different subtypes of Treg based on expression of CD25 and FoxP3. Thus, we evaluated CD25−FoxP3+, CD25+FoxP3+, CD25^Low^FoxP3+, and CD25^High^FoxP3+ cells.

**Figure 3:**
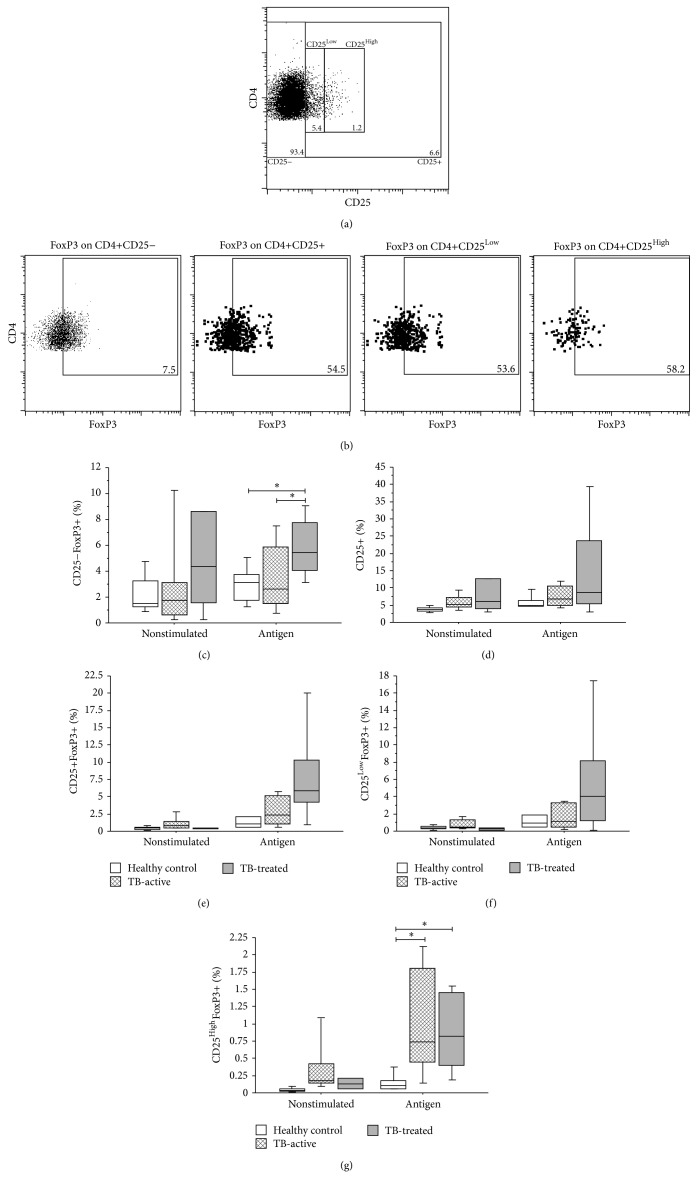
T helper regulatory cells in active tuberculosis, clinically cured tuberculosis, and TST+ healthy donors. (a) Cells were previously separated based on FSC and SSC patterns (as showed in Figure 2(a)). (b) These cells were isolated according to CD4 expression and the expression of CD25 was evaluated (upper panel). Lower left panel represents FoxP3+ cells in CD4+CD25− cells and lower right panel represents FoxP3+ cells in CD4+CD25+ cells. Positive cells were defined using an appropriate isotype control. Dot plots representative of a single participant are shown. Comparison of (c) %CD4+CD25−, (d) %CD4+CD25+, (e) %CD4+CD25+FoxP3+, (f) %CD4+CD25^Low^FoxP3+, and (g) %CD4+CD25^High^FoxP3+ among TST+ healthy donors (white box-plots), active-TB patients (dashed box-plots), and TB-treated patients (gray box-plots) in stimulated (4 *μ*g/mL* M. bovis* antigen) and unstimulated (medium only) cultures. ^*^ *P* < 0.05: Kruskal-Wallis test followed by Dunn's post hoc test. Horizontal lines represent the median, bars represent 25–75th percentiles, and vertical lines represent 10–90th percentiles.

We observed that TB-treated patients had a higher percentage of CD4+CD25−FoxP3+ than TST+ controls and TB-active patients in cultures stimulated with antigen (*P* = 0.027 and *P* = 0.019, resp., Kruskal-Wallis test followed by Dunn's post hoc test), Figure 3(c). Regarding the expression of CD25, although we observed an upward trend associated with clinical cure, no significant difference was observed, Figure 3(d).

We observed an increase in both CD4+CD25+FoxP3+ cells and CD4+CD25^Low^FoxP3+ cells in TB-treated patients compared to active-TB patients and healthy donors, Figures 3(e) and 3(f). However, an increase in the* in vitro *percentage of CD4+CD25^High^FoxP3 cells was observed in active pulmonary tuberculosis patients in stimulated cultures (*P* = 0.029 resp., Kruskal-Wallis test followed by Dunn's post hoc test). These cells remained significantly elevated in patients after clinical cure in stimulated cultures (*P* = 0.045, resp., Kruskal-Wallis test followed by Dunn's post hoc test), Figure 3(g).

### 3.4. Active-TB and TB-Treated Patients and TST+ Healthy Donors Present Different Ratios of T Helper Subsets

The overall profile of immune response can be influenced not only by the percentage of cells committed to specific phenotypes but also by the ratio of cells with different functions. Based on this phenomenon, we investigated whether differences in the percentage of* Mycobacterium*-specific T helper cells influenced the growing prevalence of effector populations (Th1, Th2, and Th17), regulatory populations (Treg subtypes), or the ratio among Th1, Th2, and Th17 cells.

**Figure 4:**
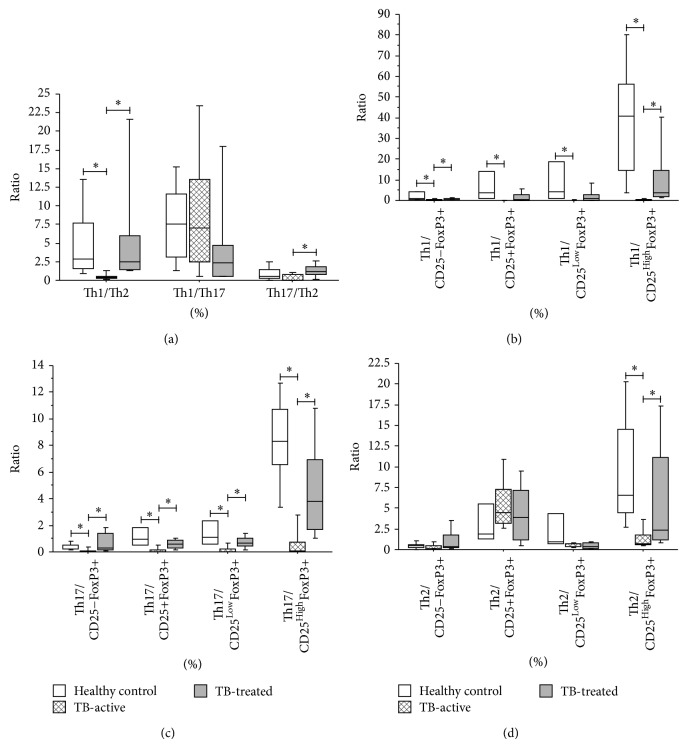
Ratios between different subtypes of* Mycobacterium*-specific T cells in patients with active tuberculosis, patients after treatment, and TST+ healthy donors. The ratios were calculated using the percentages of double positive cells (transcription factor and cytokine) in cultures stimulated with BCG antigen. (a) From left to right: Th1 (CD4+T-Bet+IFN-*γ*+)/Th2 (CD4+GATA-3+IL-4+), Th1/Th17 (CD4+ROR*γ*T+IL-17+), and Th17/Th2. (b) From left to right: Th1/CD4+CD25−FoxP3+, Th1/CD4+CD25+FoxP3+, Th1/CD4+CD25^Low^FoxP3+, and Th1/CD4+CD25^High^FoxP3+. (c) From left to right: Th2/CD4+CD25−FoxP3+, Th2/CD4+CD25+FoxP3+, Th2/CD4+CD25^Low^FoxP3+, and Th2/CD4+CD25^High^FoxP3+. (d) From left to right: Th17/CD4+CD25−FoxP3+, Th17/CD4+CD25+FoxP3+, Th17/CD4+CD25^Low^FoxP3+, and Th17/CD4+CD25^High^FoxP3+. ^*^ *P* < 0.05: Kruskal-Wallis test followed by Dunn's post hoc test. Horizontal lines represent the median, bars represent 25th–75th percentiles, and vertical lines represent 10th–90th percentiles.

We observed that healthy controls and individuals with clinically cured tuberculosis exhibit a significantly elevated Th1 : Th2 ratio (*P* = 0.003 and *P* = 0.004, resp., Kruskal-Wallis test followed by Dunn's post hoc test), Figure 4(a), left bars. Additionally, TB-treated patients have a higher Th17 : Th2 ratio than active-TB patients (*P* = 0.014, Kruskal-Wallis test followed by Dunn's post hoc test), Figure 4(a), right bars.

Regarding regulatory T cells, TST+ healthy controls had higher Th1 : Treg ratio for Treg CD4+CD25−FoxP3+ and CD4+CD25^High^FoxP3+ subpopulations than TB-active patients (*P* = 0.007 and *P* = 0.023, resp., Kruskal-Wallis test followed by Dunn's post hoc test) and Th17 : Treg ratios for all the CD4+CD25+FoxP3+ subpopulations evaluated (CD25+, *P* = 0.008; CD25^Low^, *P* = 0.015; and CD25^High^, *P* = 0.017, Kruskal-Wallis test followed by Dunn's post hoc test), Figures 4(b) and 4(c). Similar results were observed in TB-treated patients for CD4+CD25−FoxP3+ and CD4+CD25^High^FoxP3+ relative to Th1 cells (CD25−, *P* = 0.046; CD25^High^, *P* = 0.0028, Kruskal-Wallis test followed by Dunn's post hoc test) and Th17 cells (CD25−, *P* = 0.013; CD25^High^, *P* = 0.034, Kruskal-Wallis test followed by Dunn's post hoc test). Regarding the Th2 : Treg ratio, significant differences (elevated ratios) were only observed for CD4+CD25^High^FoxP3+ in TB-Treated patients and healthy controls (*P* = 0.048 and *P* = 0.016, resp., Kruskal-Wallis test followed by Dunn's post hoc test).

Taken together, our data indicate that important phenomena are related to the development of active tuberculosis and subsequent clinical cure. The process of triggering active disease appears to reflect a small but broad repertoire of T helper cells (Th1, Th2, and Th17) and an increase in CD4+CD25^High^FoxP3+ cells, compared with those in TST+ individuals without disease. Clinical cure of tuberculosis leads to a significant increase in all T cell profiles evaluated (Th1, Th2, Th17, and Treg, although with differences among subtypes of the latter). Interestingly, this repertoire is still higher than that in TST+ individuals with no history of active disease. Moreover, although they displayed a smaller overall repertoire of Th cells, active-TB patients showed a ratio of these cells favoring Th2 and Treg populations, whereas healthy controls and clinically cured patients had a higher proportion of Th1 and Th17 cells.

## 4. Discussion

Our data indicate a decreased capacity for activation of helper T lymphocytes in patients with active tuberculosis, represented by the lowest percentage of CD69+ cells. Additionally, the clinical cure of tuberculosis led to a greater repertoire of CD4+ T cells activated by soluble antigens derived from* M. bovis*, demonstrating the potential of these antigens to stimulate a specific response due to prior development of tuberculosis, contact with mycobacteria without development of disease, or BCG vaccination, which is widely administered in the study region.

Several previous reports also suggest a deficiency in this activation in active tuberculosis patients, in both CD4+ T cells and CD8+ T cells [29–31]. Our results suggest the possibility that, in patients with active disease,* Mycobacterium* antigen-specific T cells are retained in the lungs, thus decreasing the circulating population. Cured individuals, however, present a large number of specific, highly activated lymphocytes (CD69+) in peripheral circulation. Indeed, several authors demonstrate that there is not always a relationship between circulating T cell clones and those retained in an infectious site, especially in active tuberculosis [32–34]. An important aspect to be considered is whether the lower percentage of activated T cells observed in active disease patients is a consequence of tuberculosis reactivation, in which microbial antigens impair the antigen presentation and differentiation of T helper lineages, or whether this decrease is actually the primary cause of disease reactivation. In fact, it has been demonstrated that* Mycobacterium tuberculosis* molecules can negatively modulate antigen presentation, thus impacting immunoregulatory cytokine secretion [35].

The role of T helper cells has been extensively investigated in active disease and progression to clinically cured posttreatment status (for a review, see [36]). As already discussed, cellular immunity, especially mechanisms based on Th1 cells, is highly involved in the immune response to infection with* M. tuberculosis*. IFN-*γ* is the major cytokine secreted by Th1 cells, and its importance has been broadly investigated. Several experimental models demonstrate a major susceptibility to* M. tuberculosis* infection when IFN-*γ* production is compromised [4, 37]. In fact, our results point to a reduction in Th1 cells in active-TB patients followed by an increase in these cells after clinical cure. Our group also described this outcome in previous results that evaluated culture supernatants [5], in accordance with previous reports [12, 13, 29, 38–42]. Shams et al. demonstrate that these active-TB patients present a lower frequency of IFN-*γ*-producing CD4+ T cells, a result that is more evident in severe disease [43]. A recent study found a reduction in IFN-*γ*-producing cells that were associated with active disease when compared to those from noninfected controls [44]. Furthermore,* Mycobacterium tuberculosis*-infected mice lacking T-Bet display an increased susceptibility to disease, most likely due to an IFN-*γ*/IL-10 imbalance [45]. Over the years, several studies have identified genetic polymorphisms associated with different molecules in the IFN-*γ* induction pathway, especially T-Bet, STAT1, and IFNGR1/IFNGR2 [46], and these polymorphisms seem to influence susceptibility to several infectious diseases, including tuberculosis [46–48]. Recent reports have shown that STAT1 polymorphisms impair early, but not late, responses to interferons [49]. It has also been shown that the relationship between APCs and T cells appears to be compromised by these polymorphisms in the IFN-*γ* pathway, via direct influence on the induction of cellular immune response profiles [46]. These aspects could have a direct effect on the development of active tuberculosis, by delaying the recovery of IFN-*γ* production that is required for clinical cure. In fact, polymorphisms in IFN-*γ* as well as in IL-10, TGF-*β*, and IL-12 can influence the response to antituberculosis treatment [50]. Furthermore, the immune response to mycobacterial infections can be affected by epigenetic modifications, as recently demonstrated in a bacillus Calmette-Guérin infection [51], and these mechanisms have been shown to be essential for the development and stability of T helper cells [52, 53].

In human tuberculosis patients, the roles of Th2 cells and IL-4 are similarly controversial. Surcel et al. and van Crevel et al. found an association between an increase of IL-4 and active tuberculosis development, especially in severe forms of the disease [11, 54]; Lin et al. observed a decreased Th1 response in active patients who is not accompanied by an increased Th2 response [12]. Recently, Winkler et al. reported that, even in active patients presenting a decrease in IFN-*γ*-secreting CD4+ Th1 cells, there was no difference in the percentage of IL-4- or IL-13-secreting CD4+ cells when compared with TST+ controls [31], similar to our results. Recently, the participation of Th2 cytokines in susceptibility to tuberculosis has been associated with the inhibition of autophagy, which is important in the immune response against* M. tuberculosis* infection [9, 55, 56]. These findings corroborate the concept that a Th2 profile favors the development of active tuberculosis. However, it is still unclear if Th2 cytokine production is the primary cause of tuberculosis reactivation or simply a consequence of active infection [57]. In particular, our data indicate that active tuberculosis is not characterized by an increase of Th2 cells compared with TST+ Healthy controls, but Th2* Mycobacterium-*specific cells are augmented in clinically cured tuberculosis patients. However, despite presenting lower numbers of Th1 and Th2 cells than TB-treated patients, active-TB patients show a Th1 : Th2 ratio favoring CD4+GATA-3+IL-4+ cells. This reversal may be related to imbalances in the host-parasite relationship and the development of active disease, as well as to difficulties clearly determining the function of Th2 cells in the establishment and progression of pulmonary tuberculosis.

Studies in experimental models originally proposed that Th17 cells would be dispensable for protection against mycobacterial infection [58]. However, later studies showed that IL-17-secreting cells are involved in several aspects of the immune response to tuberculosis, both in human and experimental models; these Th17 cells may confer protection through IFN-*γ*-independent mechanisms by driving the initial events of granuloma formation and remain as long-lived memory cells [15, 59, 60]. Studies in humans have pointed to a deficient Th17 response in active TB patients, especially when compared with latent TB, a finding that seems to be related to Th17 cell recruitment to the lung environment [61, 62]. Despite disagreement about the phenotypic stability of Th17 cells, particularly regarding the transition to a Th1 phenotype [63–65], in a* Mycobacterium*-specific immune response these cells seem to assume a stable and distinct identity from Th1 response [60, 66]. Our study shows an induction of Th17 cells associated with clinical cure, parallel to an increase in Th1 and Th2 cells. Despite this overall increase in* Mycobacterium*-specific T cell subsets, clinically cured tuberculosis is accompanied by a functional repertoire with a favorable Th1/Th17 ratio.

These two populations seem to actively influence each other during infection with* M. tuberculosis*. Recent studies have shown that IL-17 production and the induction of neutrophil accumulation in the lungs aggravate tissue damage induced by* M. tuberculosis* infection [67], and high levels of IFN-*γ* production reduce this migration and associated lesions [68]. On the other hand, Wozniak et al. demonstrate that IL-17-secreting cells can provide IFN-*γ*-independent protection in tuberculosis, but that cross-regulation between Th1 and Th17 seems to be essential to confer a significant protective effect against* M. tuberculosis* and reduce damage to infected organs [59]. This Th1/Th17 interface is likely to be directly related to clinical cure, with control of microorganisms, but not sterilization, reducing tissue damage.

Naturally occurring CD4+CD25+ regulatory T (Treg) cells, which constitutively express the transcription factor Foxp3, are indispensable for the maintenance of immune self-tolerance and homeostasis by suppressing aberrant or excessive immune responses harmful to the host [69, 70]. The majority of Foxp3+ natural Treg (nTreg) cells are produced by the thymus as an antigen-primed and functionally mature T cell subpopulation specialized for immune suppression. Some of these cells also differentiate from naive conventional T (Tconv) cells in the periphery under certain conditions. The main task of Foxp3+ nTreg cells is to migrate to inflammation sites and suppress various effector lymphocytes, especially helper T (Th) cell subsets: Th1, Th2, Th17, and follicular Th (Tfh) cells [71–74]. Although most Treg cells are CD25+, some are CD4+CD25−FoxP3+, which reflects induced Tregs [75]. Studies of the direct effect of Treg cells in tuberculosis suggest that these cells participate in immunosuppression observed in individuals with more severe active disease [20], showing that T regulatory cells are increased in these individuals and also exercise regulatory activity, with IFN-*γ* levels returning to normal after depletion [21, 22]. Previous results suggest an important role of Tregs in reactivation of latent infection and in the development of active tuberculosis by decreasing IFN-*γ* responses, while IL-17 may continue facilitating the accumulation of cells in inflamed tissues [76]. Furthermore, a previous report points to an increase in CD4+CD25+ and CD4+CD25+FoxP3+ cells associated with active pulmonary tuberculosis [77], but the inactivation of CD4+CD25+ T cells has no effect on pathogen load and infection-induced lung pathology [78]. In addition, there are very few studies comparing T helper subsets in active disease and after clinical cure. Our results indicate that, rather than an increase in the percentage of* Mycobacterium*-specific Tregs, active TB presents lower Th1 : Treg and Th17 : Treg ratios. These data, together with lower Th1 : Th2 and Th17 : Th2 ratios, may indicate a mechanism by which the breakdown of the host-parasite equilibrium leads to active tuberculosis and that changes in the repertoire of* Mycobacterium*-specific Th cells are associated with clinical cure after treatment of pulmonary tuberculosis.
